# Current Research on Quantifying Cotton Yield Responses to Waterlogging Stress: Indicators and Yield Vulnerability

**DOI:** 10.3390/plants14152293

**Published:** 2025-07-25

**Authors:** Long Qian, Yunying Luo, Kai Duan

**Affiliations:** 1 Changjiang Institute of Technology, Wuhan 430212, China; qianlong@whu.edu.cn (L.Q.);; 2 School of Civil Engineering, Sun Yat-sen University, Guangzhou 510275, China

**Keywords:** cotton, flood, abiotic stress, climate change, crop water relation, agro-meteorology, field drainage, yield loss

## Abstract

Cotton (*Gossypium* spp.) is an important industrial crop, but it is vulnerable to waterlogging stress. The relationship between cotton yields and waterlogging indicators (CY-WI) is fundamental for waterlogging disaster reduction. This review systematically summarized and analyzed literature containing CY-WI relations across 1970s–2020s. China conducted the most CY-WI experiments (67%), followed by Australia (17%). Recent decades (2010s, 2000s) contributed the highest proportion of CY-WI works (49%, 15%). Surface waterlogging form is mostly employed (74%) much more than sub-surface waterlogging. The flowering and boll-forming stage, followed by the budding stage, performed the most CY-WI experiments (55%), and they showed stronger negative relations of CY-WI than other stages. Some compound stresses enhance negative relations of CY-WI, such as accompanying high temperatures, low temperatures, and shade conditions, whereas some others weaken the negative CY-WI relations, such as prior/post drought and waterlogging. Anti-waterlogging applications significantly weaken negative CY-WI relations. Regional-scale CY-WI research is increasing now, and they verified the influence of compound stresses. In future CI-WI works, we should emphasize the influence of compound stresses, establish regional CY-WI relations regarding cotton growth features, examine more updated cotton cultivars, focus on initial and late cotton stages, and explore the consequence of high-deep submergence.

## 1. Introduction

In the context of global climate change, extreme precipitation is increasingly frequent, exerting profound impacts on human society and natural ecosystems. Flooding and waterlogging disasters, usually resulting from excessive rainfalls, are worldwide natural disasters that are second only to droughts. In particular, agricultural waterlogging disasters impose great threats to crop production [1,2,3], especially for waterlogging-sensitive crops, such as cotton plants.

Agricultural waterlogging disasters, usually induced by heavy rains and over-irrigation, generally refer to ponded surface water and perched water tables in fields [4,5]. To control and reduce waterlogging disaster risks, it is crucial to reasonably and timely estimate the impact of waterlogging on crop yields. To this end, the vulnerability functions or curves (i.e., the relationships between crop yield (or losses) and disaster intensity) can serve as powerful tools. According to a recent worldwide investigation concerning crop vulnerability to climate-related disasters, the importance and notability of crop vulnerability under flood/waterlogging is second only to crop vulnerability under drought [6]. Moreover, in agricultural drainage practices, the vulnerability curves or functions of crops under waterlogging are convenient and efficient in assessment of waterlogging-induced crop yield losses.

Cotton is amongst the most important industrial and cash crops in the world. In more than 90 countries around the world [7], especially China [4,8,9], Australia [10,11], Pakistan [12,13], India [14], and America [15], cotton (*Gossypium* spp.) is extensively cultivated because both its maternal and filial tissues are economically valuable [16]. Specifically, cotton fiber is widely employed as the major source for the global textile industry, while cotton seeds are extensively used for feed and edible oil. The number of species in the genes *Gossypium* is about 50 [7], among which *Gossypium hirsutum* (i.e., upland cotton) and *Gossypium barbadense* (i.e., pima cotton) are the most popular species [16]. In terms of life span, although cotton has the habit of perennial growth and indeterminate fruiting, it is an annual crop, with a life span of around 210 days [17]. In addition, it is a herbaceous plant with a key habit of being poorly adapted to waterlogging conditions [11,18]. Therefore, in many important cotton-growing regions, cotton yields are severely restricted by waterlogging disasters [4,19]. For instance, in China, which is the largest cotton-consuming country, as well as the second-largest cotton-producing country [20], two of the three major cotton-producing regions, i.e., the Yangtze River cotton Belt and the Yellow River cotton Belt, suffer from severe cotton waterlogging problems [21,22,23].

Large numbers of investigations have been performed to comprehensively reveal the responses of cotton from various aspects, including plant morphology, physiology, and yield formation under waterlogging conditions [4,8,9,11,22]. Compared with physiological and morphological indicators, cotton yields are especially noticeable because they are more directly related to yield loss estimation. From the perspective of agricultural water management, establishing the relationships between cotton yields and waterlogging intensity (CY-WI relations for short hereafter) is crucial in scheduling proper drainage. To be specific, determining the reasonable amounts and timings of drainage is the key mission of cotton drainage, and the CY-WI relations serve as fundamental bases for this mission. In addition, from the perspective of disaster reduction, CY-WI relations are also of practical importance for disaster risk reduction. Specifically, after flooding and waterlogging disasters, a critical mission is to rapidly estimate the direct economic losses (also including crop losses), and the CY-WI relations can play a key role in loss estimation.

Over the past decades, by means of numerous cotton field experiments, the mechanisms of cotton in response to waterlogging stress have been well documented. Accordingly, there are some scholars performing sound reviews based on existing cotton waterlogging publications. For instance, Najeeb et al. [18] summarized previous studies to derive the mechanism of yield losses in cotton under waterlogging stress and also proposed strategies for reducing cotton waterlogging impacts. More recently, Zhang et al. [24] focused on cotton adaptation behaviors under waterlogging stress, especially summarizing three strategies for cotton to avoid waterlogging damages. Nevertheless, these cotton waterlogging reviews are mainly concentrated on the mechanisms of cotton plant behaviors in response to waterlogging, from molecular levels to physiological levels, lacking specific review work focusing on CY-WL relations. This kind of review is of practical importance because the agronomists, engineers, and policy makers are more interested in yield-relevant findings (i.e., CY-WL relations). More importantly, there are many new progresses in the research field of cotton waterlogging. As a result of increasing climate change, waterlogging-relevant coupling disasters/stresses are becoming more frequent in crops [25], and cotton responses to waterlogging are also affected by the interactive influence of coupling stresses, such as drought and heat stress [26,27,28]. In addition, high-quality crop and climate data are more accessible at a regional scale, promoting the exploration of regional waterlogging impacts on cotton yields (i.e., regional CY-WL relations). Due to these concepts, it is urgent to perform a thorough review work on CY-WI relations regarding the most recent advances, based on which future prospects can be proposed.

The objectives of the present work are: (a) to summarize previous cotton research in terms of CY-WI relations and (b) to introduce the most recent progress in this field, which mainly arises from rapid technology development and increasing climate change. The present work carefully searched and summarized the papers regarding cotton yield vulnerability to waterlogging (regarding CY-WI). Notably, this work roundly summarized the influential factors affecting CY-WI relations, and, for the first time, this review paid special attention to regional-scale CY-WI research.

## 2. Materials and Methods

To completely collect the existing literature concerning CY-WI relations, we explored public publications through several worldwide authoritative databases, including Web of Science, Scopus, and Engineering Index. There were three steps to determine our required papers:

Step 1: To refine the literature containing both cotton yield data and waterlogging intensity, we employed the following topic words in searching research: “cotton” AND “waterlogging” AND “yield”. The literature search timeframe was restricted to publications from 1970 onward. In addition, as waterlogging stress is sometimes described as “flood” or “aeration stress”, we replaced the topic word “waterlogging” with “flood” or “aeration stress” and performed research searching again.

Step 2: A subset of the literature we retrieved in Step 1 failed to comply with our specifications for establishing CY-WI relation. Therefore, we further screened out the collected literature according to the following standards:

(1) It must contain cotton yield data or yield loss data. Therefore, much of literature that merely reported other crop indicators (physiological or morphological indicators) rather than yields was not included.
(2) It must include indicators describing waterlogging intensity
(3) It must contain different levels of waterlogging intensity, which is the basis for CY-WI relations.

Step 3: In a few cases, different publications corresponded to the same experiments; thus, we regarded them as only one CY-WI experiment. In addition, a few regional-scale cotton investigations that employed cotton climatic yields and meteorological indicators were also included, but they are individually analyzed and discussed in later section. Eventually, we screened out 51 published experimental works that included paired data of cotton yield vs. waterlogging indicators.

In comparing the CY-WI relations among different growth stages, as cotton yield loss in different study cases can vary greatly, we screened out four experimental works containing waterlogging treatments at multiple stages and established their CY-WI relations; in this way, the comparison among different growth stages is fair and reasonable. In addition, in demonstrating the potential influence of accompanying high temperatures on CY-WI relations, we used the data collected from three cotton waterlogging publications, including normal temperature and high-temperature conditions [29,30,31], thus establishing CY-WI relations with and without accompanying high temperatures.

## 3. Current Research Progress of CY-WI Relations

Cotton waterlogging can be embodied in different forms. In public understanding, waterlogging usually refers to ponded surface water. However, perched water tables can also induce excessive soil moisture and poor soil aeration. Particularly, in some cotton-producing areas, such as the Jianghan Plain area in China, perched water tables are commonly seen; thus, local researchers often distinguished subsurface waterlogging (without ponded water) from surface waterlogging (only with perched water tables) [4,31,32,33]. To this end, in this review, both the two waterlogging forms are considered.

**Figure 1:**
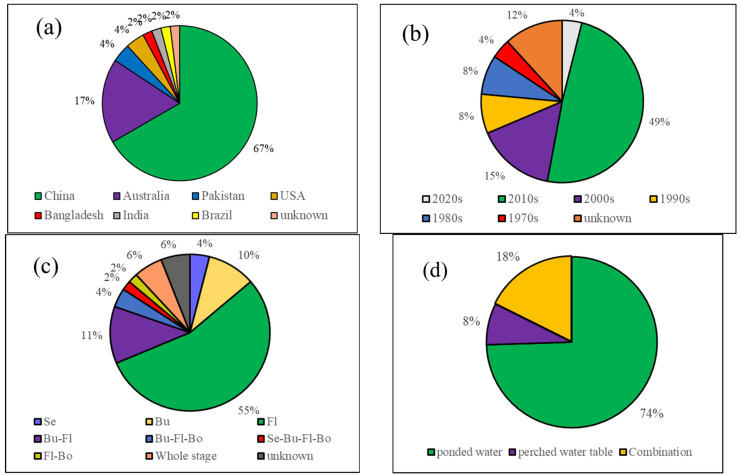
Categories of the existing CY-WI-related publications in terms of different countries (**a**), decades (**b**), examined cotton growth stages (**c**), and employed waterlogging forms (**d**). In (**c**), Se, Bu, Fl, and Bo refer to the seeding stage, budding stage, flowering and boll-setting stage, and the boll-opening stage, respectively. In (**d**), ponded water and perched water table indicate surface waterlogging and sub-surface waterlogging, respectively; combination indicates that both of the two waterlogging forms were established.

Figure 1 shows a round category of these collected experimental literatures from different perspectives. In addition, Table 1 displays some representative CY-WI experimental studies, which established various intensities of cotton waterlogging.

In terms of experiment regions (Figure 1a), previous CY-WI investigations were mostly conducted in China, accounting for 67% of total experimental works. Following China, in Australia, many trials were performed to explore local cotton yield responses to waterlogging (accounting for 17%). Some other countries, including the USA, Pakistan, India, Bangladesh, and Brazil, also contributed a few works.

In terms of experimental decades (Figure 1b), it is detected that almost half of CY-WI trials were conducted in the 2010s, followed by the 2000s (15%). Considering that the 2020s have not yet passed the halfway mark, the 2010s and 2000s can represent the near term. Therefore, it is concluded that the CL-WI works have received increasing attention during recent decades.

In terms of the examined waterlogging stages (Figure 1c), the flowering stage was examined in more than half of previous works (55%), followed by the “flowering plus budding stages” (11%) and the budding stage (10%). This is mainly because these two stages are reproductive phases for cotton plants, and they often encounter precipitation-intensive months. In comparison, there is much less exploration focusing on the initial stage (seedling) and the late stage (boll-opening).

Finally, in terms of waterlogging forms (Figure 1d), surface waterlogging was employed in most CY-WI experiments (74%), followed by the combination of surface waterlogging and sub-surface waterlogging (18%). In comparison, previous CY-WI insufficiently focused on the impact of the perched water table, which is invisible but has non-negligible influence.

## 4. Indicators for Quantifying Cotton Waterlogging

Differing from soil drought conditions, in most waterlogging cases, soil water conditions reach saturation; as a result, soil water content (at root layers) cannot reflect the different impacts under different water tables. This is the main difference between cotton drought indicators and cotton waterlogging indicators [28]. The commonly used cotton waterlogging indicators are generally derived from waterlogging durations and waterlogging depths.

### 4.1. Waterlogging Duration

Among the existing experimental studies on cotton waterlogging, gradient levels of waterlogging durations are mostly employed to establish different waterlogging treatments; accordingly, water depths/tables are often fixed at certain values. It is the most convenient way to establish diverse intensity of waterlogging.

### 4.2. Waterlogging Depth

As abovementioned, the waterlogging depths were usually fixed in experiments, and the waterlogging durations were varied. However, the consequences of different waterlogging depths on cotton yields cannot be neglected. For cotton, in addition to standing water depths, groundwater depths can also affect cotton growth and yields, to a large extent, by generating excessive soil moisture in root layers [31,35]. Although there is very limited CY-WI research focusing on subsurface waterlogging only (only 8%, Figure 1d), the combinations of surface waterlogging and subsurface waterlogging were often investigated in many works (18%, Figure 1d).

In fact, as early as the 1980s, the influence of different groundwater tables on cotton yields has been examined in many experimental studies [36,37]. However, in recent years, cotton waterlogging experiments tend to employ fixed water tables. A possible reason is that different water tables are not easily established, which requires water table-adjustable facilities. In addition, current research focuses more on the mechanisms of cotton plants; thus, the employed waterlogging form is not that important, and controlling water durations are enough.

The previous CY-WI research regarding water tables primarily explored the impacts of different groundwater depths, with rare consideration of the impacts of different surface water depths. Moreover, in most studies, the surface waterlogging depths are fixed to shallow depths (i.e., 1~20 cm). High-depth surface water was not considered, probably because cotton is easily subjected to crop lodging and can even die under deep ponded water. Consequently, to the best of our knowledge, the real effect of surface water depths on cotton yield and survivor rate is still unclear.

### 4.3. Waterlogging Depth × Waterlogging Duration (SEW_30_/or SFEW_30_)

Based on waterlogging durations and waterlogging depths, more comprehensive waterlogging indicators have been constructed to quantify the overall impact of waterlogging over a particular calculation period. The most representative indicator is the sum of the excessive water within ~0–30 cm soil profiles (SEW_30_) [38], which is calculated as follows:

(1) $${SEW}_{30}=\sum_{i=1}^{n}\left(30-D_{i}\right)$$

where D_i_ is the groundwater table depth on day i, and n is the total days of the calculation period.

This comprehensive indicator is essentially the product of waterlogging duration and waterlogging depth and has been commonly used in field drainage engineering. In particular, the SEW_30_ has been integrated into the famous agro-hydrological model DRAINMOD as the crop module [39,40]. For cotton plants, the SEW_30_ is also frequently employed to quantify sub-surface waterlogging intensity [4,41,42]. This indicator is suitable for characterizing cotton waterlogging because cotton root layers under water stresses are mainly concentrated in the ~0–30 cm soil profile [43].

Based on the SEW_30_, an improved comprehensive indicator, namely the sum of the flooding water depth and the excessive water-table depth with ~0–30 cm soils (SFEW_30_), has been put forward to additionally account for surface water impacts [44]:

(2) $${SFEW}_{30}=\sum_{i=1}^{n}H_{i}+\sum_{i=1}^{n}\left(30-D_{i}\right)$$

where H_i_ is the surface water depth on day i.

The SEW_30_ and SFEW_30_ are preferable to individual waterlogging durations or waterlogging depths; thus, they have been widely applied in China [5,26,31,32]. In addition, the SEW_30_ and SFEW_30_ can be conveniently associated with drainage schedules, thus being an excellent choice for cotton waterlogging assessment [45,46].

### 4.4. Improved Indices Based on SEW_30_/SFEW_30_

If the overall impact of waterlogging over the whole growing seasons is computed, the SEW_30_/SFEW_30_ cannot reflect the differences between crop growth stages. Thus, the Stress Day Index (SDI) was constructed to account for the growth-stage effect in applying the SEW [40]. In the SDI, yield losses at different growth stages are determined, and their normalization values are used as modified coefficients to construct a comprehensive indicator for waterlogging impact on crops over the whole growing season. To the best of our knowledge, the SDI for cotton was rarely established. However, under the inspiration of SDI, a more comprehensive indicator called cumulative SFEW_30_ (CSFEW_30_) has been developed to quantify cotton waterlogging over cotton seasons [47]. Just like the SDI, the CSEFW_30_ also considers the growth-stage effect of cotton waterlogging. Moreover, it further considers the different yield-reducing effects of surface waterlogging and sub-surface waterlogging because cotton yield losses reduced by surface waterlogging were much greater than those by sub-surface waterlogging [4]. Finally, the CSFEW_30_ and its corresponding CY-WI relation are expressed as follows:

(3) $${CSFEW}_{30}=\sum_{i=1}^{m}{CS}_{i}\times \left(S_{i}+k_{W}\times W_{i}\right)$$

(4) $$Rd=0.122\times CSFEW\;(n=15,\;R^{2}=0.629,\;p<0.001)$$

where CS_i_ is the crop sensitivity coefficient for stage i. S_i_ and W_i_ represent submergence intensity and waterlogging intensity during stage i, respectively. k_W_ is the modifying coefficient for transferring waterlogging intensity to submergence intensity. However, it should be noted that the CSFEW_30_ method requires a set of experienced parameters (e.g., CS_i_ and k_W_ at different stages) that are calibrated by local data, restricting its wide application.

### 4.5. Regional-Scale Indicators in Establishing CY-WI Relations

**Figure 2:**
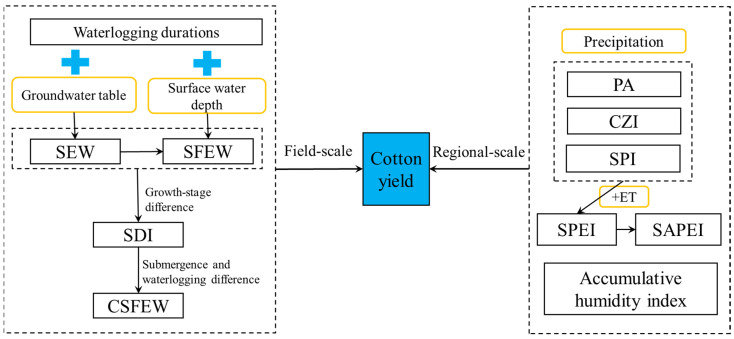
Current indicators employed in establishing CY-WI relations at both field and regional scales. SEW is sum of excess groundwater tables. SFEW is sum of flooding depth and excess groundwater tables. PA is precipitation anomaly, SPI is standardized precipitation index, SPEI is standardized precipitation and evapotranspiration index, SAPEI is standardized antecedent precipitation and evapotranspiration index.

In the abovementioned field of waterlogging indicators, water tables are the most fundamental data, but they are not easily accessed at large scale. Due to the rapid development of observation and monitoring technology, regional-scale research has promoted the progress of many research fields, including crop waterlogging monitoring. Consequently, the relations between crop yields and corresponding waterlogging disaster intensities are more frequently established at a regional scale. Compared with field-scale waterlogging indicators, regional waterlogging indicators for cotton usually employed precipitation-based meteorological indicators, which are widely accessible. So far, for cotton plants, waterlogging intensity during growing seasons have been characterized by the PA (precipitation anomaly), the SPI (standardized precipitation index), the SPEI (standardized precipitation and evapotranspiration index), the SAPEI (standardized antecedent precipitation and evapotranspiration index), and the AHI (accumulative humidity index) [2,48,49,50], and these indicators’ connections to cotton yield variations were also explored; that is, CY-WI relations at regional scales. Figure 2 summarizes current indicators employed in CY-WI relations to characterize waterlogging intensity at both field and regional scales.

## 5. Forms of Relationships Between Waterlogging and Cotton Yield

In common sense, increased waterlogging intensity results in lower cotton yields. Therefore, the linear relations of waterlogging intensity vs. cotton yields are most commonly established. Although linear CY-WI relations are simple and popular, the non-linear forms seem more suitable [2]. For instance, in an early cotton experimental study [34], the relationships between cotton lint yields and flooding hours were more significant in quadratic form (*p* < 0.01) than in linear form (*p* < 0.05). Moreover, the quadratic curves indicated that waterlogging-induced yield losses would be fewer when waterlogging intensity was severe enough. In addition, according to the most recent cotton research by Beegum et al. [15], the nonlinear forms, namely exponential decay function, were the best descriptive forms for the relations between cotton dry matter weight and waterlogging indicators.

Furthermore, more comprehensive process-oriented approaches have been developed to quantify cotton yield responses to waterlogging. For instance, the Morgan model [51], a daily-step dynamic yield-response model available for drought stress, has been revised by integrating the SFEW_30_ to simulate cotton dry matter responses to waterlogging; the revised Morgan model obviously outperformed the linear CY-WI relation that directly related the SFEW_30_ to cotton yields [52]. This dynamic CY-WI relation in the revised Morgan model is as follows:

(5) $$DY=DY0\times \prod_{t=1}^{m}{\tau (t)}^{\sigma_{SFEW30}}$$

where DY and DY0 are final and initial dry matter yield, respectively. m is the growth stage number. г(t) is the growth proportion of dry matter at growth stage t to that of growth stage (t − 1). σ_SFEW30_ is the modifying coefficient that employs the SFEW_30_ to express the influence of waterlogging stress on cotton dry matter growth.

Afterward, to establish a more dynamic mechanism model for cotton yield-response simulation under waterlogging, Qian et al. [45] adapted a physical-based dynamic yield response model, namely CROPR [53], to waterlogging conditions by replacing the soil water suction with the SFEW_30_. The improved CROPR model exhibited satisfactory performance, especially outperforming the original model. The dynamic CY-WI relations in the improved CROPR model are as follows:

(6) $$DY=DY0\times \sum_{i=1}^{n}\left(1+\frac{\sqrt{{\left(A\times w+G_{t}\right)}^{2}-3.96A\times w\times G_{t}}}{2{DM}_{i-1}}\right)$$

where n is the number of cotton growth days. DM_i_ is the actual dry matter yield on day i. A is an undetermined coefficient named the maximum efficiency of soil aeration. w is soil aeration input, which is calculated by the SFEW_30_. G_t_ is the growth amount of cotton dry matter on day i, which can be computed with solar radiation flux and other crop parameters.

## 6. Influential Factors in CY-WI Relations

### 6.1. Growth-Stage Effect of Cotton Waterlogging

**Figure 3:**
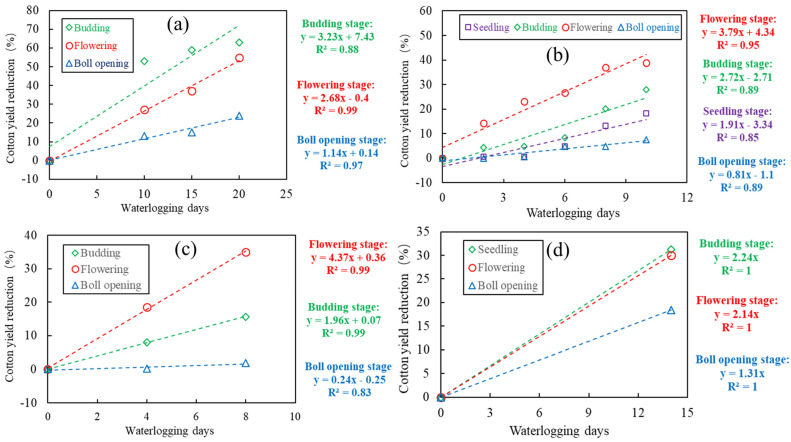
The CY-WI relations at different cotton growth stages of four cotton waterlogging experiments. (**a**–**d**) are constructed by using the data collected from Zhang et al. (2016) [22], Wang et al. (2017) [23], Qian et al., (2015) [4], and Zhu et al. (2002) [41], respectively.

**Figure 4:**
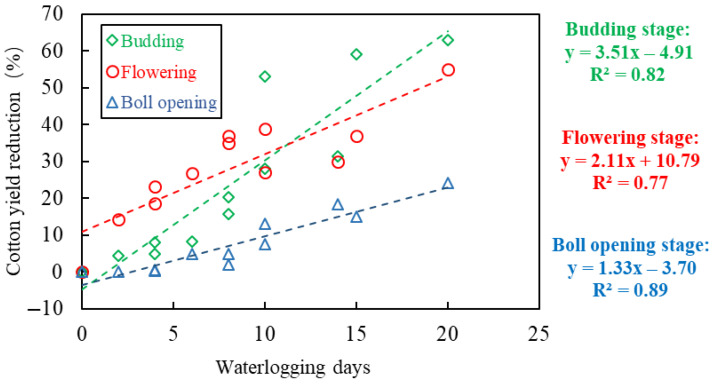
Th CY-WI relations for the budding stage, the flowering and boll-forming stage, and the boll opening stage, based on data from different multi-stage cotton waterlogging experiments, including Zhang et al. (2016) [22], Wang et al. (2017) [23], Qian et al., (2015) [4], and Zhu et al. (2002) [41].

For the same cotton plant, the CY-WI relation can be totally different among different growth stages. Figure 3 shows the results of four CY-WI trials, including multiple growth stages. CY-WI relations varied greatly with growth stages. Generally speaking, for the initial stage and the late stage (i.e., seeding and boll-opening), the CY-WI relations always correspond to the least waterlogging sensitivity of cotton; in comparison, the CY-WI relations at the budding stage and the flowering and boll-forming stage correspond to much greater waterlogging sensitivity. However, in Figure (a), the CY-WI relation with the greatest slope is in the budding stage, differing from Figure (b) and (c), where the CY-WI relation with the greatest slope was found in the flowering and boll-forming stage. This discrepancy is probably because different specific waterlogging timings can hardly be described by different stages, and cotton in different experiments exhibited different waterlogging tolerances. Furthermore, based on these four experiments, we constructed CY-WI relations for the budding stage, the flowering and boll-forming stage, and the boll-opening stage, respectively (see Figure 4). According to the overall results across these cotton waterlogging experiments, the budding stage has the most negative relations between cotton yield and waterlogging intensity, followed by the flowering and boll-forming stage. Moreover, all of these experiments prove that CY-WI relations should be individually established for different stages.

### 6.2. Cotton Genotypic Variation

Genotypic variation, which determines crops’ resistance to abiotic stress, is a non-ignorable factor in crop yield responses to waterlogging stress. In previous CY-WL works, the employed cotton variations are always determined by local cultivation traditions. Consequently, in most works, a single cotton variety was employed, which has become a source of variations in different experimental results. There are a few works [10,12,13] specifically focusing on the waterlogging tolerances of different cotton varieties. Table 2 shows a summary of previous CY-WI relations with multiple cotton cultivars in terms of the influence of cotton variations on CY-WI relations.

Hussain et al. [12] examined the waterlogging tolerance of up to 60 cotton variations by comparing their survival percentages. When these cotton varieties were subjected to 14-day waterlogging stress during the budding stage, 5 of them had survival percentages of ≤30%, 32 of them were ~31–60%, and the rest 23 were more than 60%. This finding vividly demonstrated the potential impacts of cotton variety selection. In addition, Zhang et al. [55] established 7-day waterlogging stress to compare different cotton varieties collected from three major cotton-growing regions in China. They found that the most waterlogging-tolerant cotton variety was found in the Yangtze River region, with yield losses of 7.6% and 4.7%, respectively. In comparison, the most waterlogging-vulnerable variety was found in the Inland Northwest, China, with yield losses of 21.8% and 15.5%. Thus, it can be deduced that the CY-WI relations can be quite different for different regions, even in the same country.

In a summary, it is of practical importance to determine the CY-WI for cotton varieties with different waterlogging tolerance, since planting waterlogging-tolerant crops in waterlogging-prone areas is an important strategy to adapt to climate change and reduce the impacts from crop waterlogging [56].

### 6.3. Cotton Waterlogging-Relevant Compound Stresses

**Figure 5:**
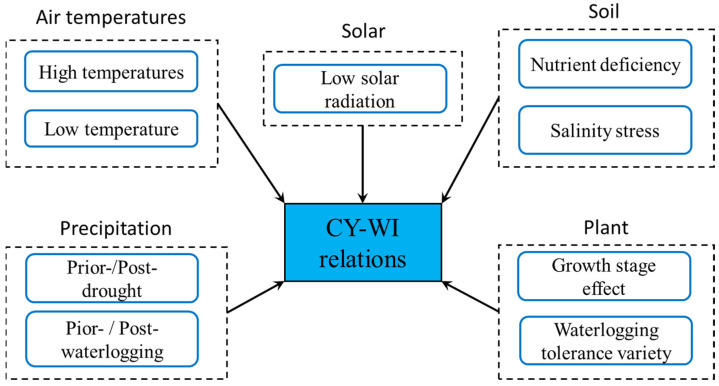
Influential factors on CY-WI relations.

Cotton waterlogging stress is mainly the result of flooding disaster, a commonly seen climatic disaster worldwide. Nevertheless, cotton plants have to face a variety of other disasters, which can occur simultaneously or sequentially with waterlogging disasters. Consequently, the occurrence of these compound disasters/stresses inevitably affects CY-WL relations. Among current published works regarding CY-WI relations, compound disasters/stresses are primarily induced by air temperatures, precipitation, solar radiation, and soil stress (Figure 5).

#### 6.3.1. The Additional Influence of Prior or Post-Drought Events

Under increasing climate change, drought and flooding abrupt alternations frequently occur. In many regions, such as Yangtze River cotton belt, cotton production is severely affected by drought and flooding abrupt alternation [17,28]. According to experimental evidences, drought occurring prior-waterlogging [27] or post-waterlogging [28] can greatly alleviate the adverse impacts of waterlogging on cotton yields; that is, cotton drought and waterlogging events have significant interactive influences. Therefore, prior/post droughts can affect CY-WI relations. Moreover, this field-scale conclusion has been verified by a regional cotton study [17]. Also in this publication, it indicated that the alternations of cotton drought and waterlogging alternations witnessed increasing trends. Thus, in the practical usage of CY-WL relations, the CY-WL relations should account for this interactive influence in the future. However, it is still very challenging to quantify the influence of drought occurrence on the yield-reducing impact of cotton waterlogging, which calls for more experimental works specifically designing for alternation of drought and waterlogging.

#### 6.3.2. The Additional Influence of Accompanying High Temperatures

In many cotton-producing regions, cotton plants are prone to accompanying high temperatures during waterlogging periods [57,58]. Qian et al. [5] employed the data from multi-year and multi-site cotton waterlogging experiments to establish structural equation models to quantify the relations between cotton growth and yields and waterlogging intensity. They found that high temperatures occurring during cotton waterlogging periods significantly increased cotton yield vulnerability under waterlogging stress; as a result, CY-WI relations were obviously affected by this accompanying factor. This phenomena can be demonstrated vividly in another publication [4]; the yield-reduction rates (i.e., slope of linear regression for CY-WL relations) during the cotton flowering stage significantly (*p* value = 0.07) increased with the corresponding maximum air temperatures. In addition, Wu et al. [29] conducted a temperature-controlled cotton waterlogging trial that considers multiple levels of waterlogging durations and accompanying high temperatures. Their results indicated that cotton yield losses, from high to low, are waterlogging and high temperature, individual waterlogging, and individual high temperature, respectively. Notably, they accordingly made suggestions for cotton drainage; that is, if accompanying high temperatures lasted for 4 days, the waterlogging water should be drained out in 3.4 days to prevent a critical cotton yield loss of 20%. A most recent study [26] proposed a dynamic yield-response model for cotton under waterlogging and accompanying high temperatures. According to their model simulations, the CL-WL relations under normal temperatures are not suitable for high temperature conditions. That is, in determining cotton field drainage standards, we should distinguish the CY-WI relations under high temperatures (>35 °C) and without high temperatures; the required drainage time for high-temperature conditions will be obviously shortened as compared with normal temperature conditions.

**Figure 6:**
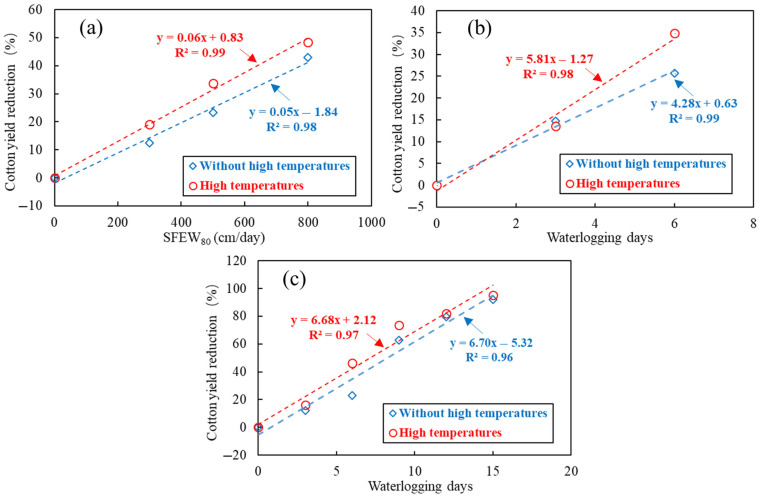
The CY-WI relations under waterlogging without accompanying high temperatures and waterlogging with accompanying high temperatures. (**a**–**c**) are constructed by the data collected from [29,30,31], respectively.

In Figure 6a,b, the slopes of CY-WI relations with high temperatures exceed those of CY-WI relations without temperatures. By comparison, in (c), the CY-WL relations seem not affected by high temperatures. This is probably because in summer seasons, the temperature difference between the waterlogging treatments and the waterlogging plus air temperature treatments was not enough.

#### 6.3.3. Interaction Among Consecutive Waterlogging Events

In many CY-WI experiments, the established waterlogging treatments were idealized; that is, cotton plants were assumed to suffer individual waterlogging events. However, a more practical situation is that waterlogging events usually occurred multiple times during rainy seasons. For instance, in the middle-and-lower reaches of Yangtze River (an important cotton-producing area in China), cotton grows from April to November, coinciding with the major rainy months; as a result, local cotton plants tend to experience waterlogging events consecutively at various growth stages [59]. In conclusion, multiple waterlogging events are more practical situations, but the interactive effect among different waterlogging events has been rarely explored.

A previous report [60] showed that the cotton yield loss induced by surface waterlogging followed by sub-surface waterlogging was obviously less than the sum of the yield losses by the two individual waterloggings. This is because the early waterlogging stress strengthened the tolerance of cotton plants to late waterlogging events. Although cotton is sensitive to waterlogging stress, its root systems have adaptive behaviors under waterlogging, especially the rapid growth of small roots after waterlogging [61]. Therefore, for multiple waterlogging events, former ones probably make cotton plants more tolerant to late ones, which also affects the determination of CY-WI relations.

#### 6.3.4. The Additional Influence of Accompanying Low Temperatures

Although cotton is generally cultivated in warm regions, sometimes low temperatures can also occur during cotton waterlogging periods. Zhang et al. [62] experimentally examined the impairs of the coupling of cotton waterlogging and low temperatures, and they compared four experimental treatments, including non-waterlogging and normal temperature, non-waterlogging and low temperatures, waterlogging and normal temperatures, and waterlogging and low temperatures. Seed cotton yields of these four treatments were 48.6, 31.1, 35.1, and 27.4 (g/plant), respectively. Therefore, the occurrence of low temperatures would enhance cotton yield reduction under waterlogging conditions.

#### 6.3.5. The Additional Influence of Accompanying Shade Conditions (Low Solar Radiation)

Cotton waterlogging conditions are primarily induced by rainy weather during cotton growth seasons. Low-incident light is also harmful to cotton growth. Thus, it is suggested that CY-WI relations may be affected by the additional influence of low-incident light (shade conditions) during cotton waterlogging periods. According to a previous cotton experiment, low solar radiation can significantly exacerbate yield losses under cotton waterlogging, but in the conditions of modest waterlogging [63].

#### 6.3.6. The Additional Influence of Soil Nutrient and Salinity

Soil nutrients are important inputs for cotton growth and yield formation, and insufficient soil nutrients can affect cotton yield in response to waterlogging. As clearly demonstrated in a previous trial [64], the reduction in dry boll matter was 13% under high soil nitrogen conditions, but it reached 48% under low nitrogen conditions. Therefore, soil nitrogen deficiency can influence CY-WL relations.

Waterlogging and salinity stresses often occur synchronously in some regions. According to a field experiment concerning waterlogging and salinity stress [65], waterlogging (with groundwater depth < 1 m) can result in 60% yield loss, and this reduction increased with the accompanying soil salinity. In particular, when the soil salinity > 12 dS/s, the un-waterlogged cotton plants had a yield of 149 kg/ha, but the waterlogged cotton had no yields. Therefore, for the areas with soil salinity stress, the establishment of CY-WL must account for this influence.

### 6.4. The Influence of External Anti-Waterlogging Applications

In addition to the abovementioned coupling disasters, there are other external human factors that affect CY-WI relations, i.e., anti-waterlogging applications. Table 3 presents current anti-waterlogging applications employed in CY-WI research. Since these measures are taken to relieve cotton waterlogging impacts, their influences on CY-WI relations are always significant, i.e., weakening the negative relations of cotton yields and waterlogging intensity.

Applying nitrogen before waterlogging is considered as a critical measure to alleviate crop waterlogging stress [66]. In addition to soil nitrogen application, foliar-applied nitrogen is also used to increase cotton tolerance to waterlogging [67,68]. As early as the 1980s, the foliar-applied nitrogen was examined in ameliorating waterlogging damage to cotton [67], and this method proved to be effective in most cases. In a recent trial concerning waterlogging nitrogen [69], for 5-day and 10-day cotton waterlogging, the application of 240 and 360 kg N/ha can mediate cotton yields. In addition, Li et al. [70] sprayed fertilizers and plant growth regulators on cotton crops after waterlogging, and found that these applications can reduce waterlogging-induced cotton yield losses by 28.57%. In addition to nitrogen, another important nutrient element, i.e., Potassium, was recently examined to have the efficiency to alleviate the negative impact of waterlogging on cotton growth and yield [68].

Applying exogenous anti-waterlogging chemicals can help enhance cotton resistance to stressed conditions, also including cotton waterlogging stress. A variety of anti-waterlogging chemicals, such as nitric oxide [71,72], melatonin [73], and ethylene signal transduction inhibitor [74], have been examined, and their effectiveness is significant (Table 3), implying that the negative relations of CY-WI can be obviously weakened by them.

**Table 3:** The influences of anti-waterlogging applications on cotton yield responding to waterlogging.

Influential Factors	Literature Sources	Effectiveness Description
Foliar-applied nitrogen	Hodgson and MacLeod, 1987 [67].	Foliar nitrogen applied just before waterlogging (lasting 4, 8, 16, and 32 h) can increase cotton yield by 2.5, 5.9, 8.4, and 10.5 kg/ha.
Foliar-applied nutrient regulators (brassin and diethyl aminoethyl hexanoate)	Jiang et al., 2013 [68]	By applying nutrient regulators, cotton yield losses induced by 10-day waterlogging stress can be considerably reduced from 42% to 8%.
Soil nitrogen	Guo W.Q. et al. 2010 [66].	Under 0, 240, and 480 kg/ha^2^ levels of soil nitrogen application, cotton yield reduction rates that induced by 8-day waterlogging stress were 18.2%. 25.4%, and 42.12%.
	Qi and Wu, 2023 [69]	Under 5-day waterlogging, applying a nitrogen rate of 240 kg N/ha provided a comparable yield to applying a nitrogen rate of 360 kg N/ha. A high nitrogen rate ameliorated the adverse effects of 10 d waterlogging on cotton yield.
Potassium	Huang et al., 2023 [68]	With potassium, the number of cotton bolls under 6-day waterlogging increased by 16.17%, compared with 6-day waterlogging under no potassium.
Ethylene signal transduction inhibitor (1-MCP)	Liu et al., 2020 [74]	By applying 1-MCP, the cotton yield under 10-day waterlogging stress increased by 8%.
Anti-ethylene agent aminoethoxyvinylglycine (AVG)	Najeeb et al. 2016 [75]	By applying AVG (125 g [ai] ha^−1^), cotton yield under WL was significantly increased by 13%.
Post-waterlogging soil fertilization	Wu et al. 2012 [76]	When increasing fertilization amount from normal level (210 kg/ha^2^ N and 191.25 kg/ha^2^ K_2_O) to 140% normal level, the waterlogging-induced relative cotton yield increased from 72.7% to 89.9%.
Oxygen slow-release fertilizer	Xia et al., 2016 [77]	By applying oxygen slow-release fertilizer before waterlogging, the relative cotton yield was increased from 43.31% to 50.94%.
Pirformaspore indica	Yang et al., 2015 [78]	By applying Pirformaspore indica after waterlogging, the cotton yields under 14-day waterlogging can be increased from 2453.27 to 3125 kg/ha^2^.
Melatonin	Zhang et al., 2024 [73]	By applying melatonin, the cotton yield reduction rate under 10-day waterlogging can be reduced from 46.6% to 39%.
Nitric oxide	Zhang et al. 2021, 2022 [71,72]	By applying sodium nitroprusside (SNP), the cotton yield reduction rate can be reduced from 36% to 28.4%.

## 7. Developing Regional-Scale CY-WI Relations

Currently, CY-WL relations are primarily established through field experimental data, which assist in assessing cotton yield losses due to waterlogging, thus reasonably scheduling cotton drainage. On the other hand, with the rapid development of computer technology and meteorological monitoring, there are increasingly regional-scale research assessing the impacts of climatic disasters (such as waterlogging) on crop production. For cotton plants, in addition to large amounts of field-scale investigations, the CY-WL relations have been also explored at the regional scale. Regional-scale CY-WL relations are essentially the relationships between cotton yield variations (often expressed as climatic yields) and regional meteorological indicators of waterlogging intensity over cotton growing seasons. It should be noted that the purpose and functions of regional CY-WI relations and field-scale CY-relations are quite different. As mentioned above, the field-scale CY-WI relations can benefit field drainage schedules. As for regional-scale CY-WI, they can provide guidance for policy makers by identifying high-risk periods and high-risk regions calling for efficient cotton field drainage [49,79]. Moreover, for some waterlogging-relevant compound events in cotton plants (e.g., drought-waterlogging alternation and waterlogging-heat coupling [17,57]), only by identifying the specific disaster-prone regions can we make reasonable schedules to adapt cotton drainage. In addition, with the help of future climate models, we can optimize the planting areas of cotton by growing more plants in the areas with weakened CY-WI relations, since weakened relations indicate the influence of waterlogging on cotton yields is not severe.

At regional scales, waterlogging depth data are usually hard to observe and collect. Instead, meteorological indicators (especially precipitation) are quite convenient due to their high attainableness and convenience. Moreover, large amounts of historical or future meteorological data can compute these indicators for robust analysis and simulation. Liu et al. [50] employed precipitation anomaly and crop water demand deviation as water conditions indicators and related them to cotton climatic yields in Jingzhou, Hubei Province, China; they detected significant and negative CY-WL relations during the flowering stage. In a recent study [2], up to six indicators were compared in terms of their performances in describing the yield-reducing effect of waterlogging on crop production, including cotton yields, in the middle–lower Yangtze River. Their result indicates that the CY-WI relations were found to be significantly negative in many districts, especially when using the SPEI. In another study revealing the contributions of climatic factors to cotton yield variability in China [20], the relation between precipitation and cotton yields is derived for different sub-regions in China and concludes that excessive precipitation caused 3.92% cotton yield losses in the Middle-and-Lower Yangtze River Region. More importantly, Hubei Province and Tianjin city had negative and significant CY-WI relations.

**Figure 7:**
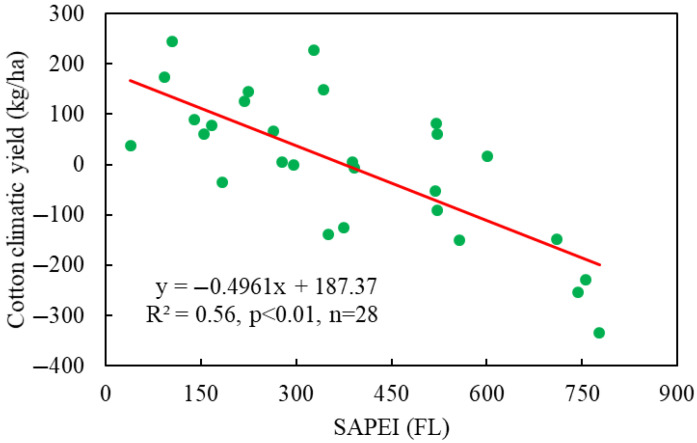
The relationship between cotton climatic yield and waterlogging intensity (characterized by the SAPEI) from 1990–2017 in Hubei Province of China.

The above works directly establish the relationships between cotton yields and meteorological indicators. Furthermore, some works integrated the SEW/SFEW concepts (Equations (1) and (2)) into meteorological indicators, constructing regional-scale waterlogging indicators to describe cotton waterlogging. Qian et al. [4] proposed two indicators, i.e., the sum of excessive precipitation (SE-P) and the sum of excessive SPEI (SE-SPEI) over cotton growth stages, to characterize cotton waterlogging disasters in Hubei Province, which is an important cotton-producing area in China; they found that the relations between cotton climatic yields and both accumulative indicators were significantly negative, and the relations were more significant when using the SE-SPEI. Later, two more comprehensive indicators, i.e., the sum of SAPEI (SE-SAPEI) [49] and the sum of accumulative humidity index [49], have been employed to reveal cotton waterlogging disasters in Hubei Province of China. Their results consistently concluded that the CY-WI relation at certain cotton growth stages was significantly negative, indicating significant cotton yield reductions by waterlogging. For a more vivid understanding, we construct Figure 7, displaying the relationships between SE-SAPEI and cotton climatic yield in Hubei Province from 1990–2017. It is clearly seen that even at regional scale, the CY-WI relations can be very significant, implying the feasibility of regional CY-WI explorations. Furthermore, in a most recent study by Tang et al. (2025) [80], based on the SE-SAPEI, a regional-scale crop waterlogging indicator named RSDI (regional stress day index) was developed, and it was applied to characterize cotton waterlogging intensity and its negative impacts on cotton yields in the Middle-and-Lower Yangtze River Region.

Since regional CY-WI relations have obvious differences to traditional field CY-WI relations, we have created a comparison table to distinguish them (Table 4). Although both of them describe the relationships between cotton yield and waterlogging indicators, they employed different waterlogging indicators, yield indicators, relation forms, and study periods.

As mentioned above, the accompanying high-temperature events during cotton waterlogging periods can alter the CY-WI relations. Moreover, according to a most recent study in the Middle-and-Lower Yangtze River Region of China [57], this additional impact from accompanying high temperatures has been confirmed at the regional scale. In that research, almost all the study areas witnessed negative CY-WI relations. More importantly, in the cases with more frequent accompanying high temperatures, the CY-WI relations would be more significantly negative.

As abovementioned, field evidences indicate that prior-waterlogging or post-waterlogging droughts can significantly weaken the CY-WI relations. This finding has also been confirmed by a recent regional investigation regarding cotton drought and flooding disasters [17]. In that work, it was detected that the abrupt alternations of drought and waterlogging can obviously weaken the negative impacts of drought and flooding on cotton yields.

## 8. Current Research Limitations and Future Prospects

### 8.1. Waterlogging-Relevant Coupled Disasters

Nowadays, the most profound feature in CY-WI relations is brought by increasing climate change. The increasing unevenly distribution of precipitation and heat result in many coupling disasters, posing new challenge for cotton production. Thus, compound disasters deserve more attention, and cotton plants are objectively subjected to a variety of flooding-relevant coupling disasters. These factors can significantly influence the efficiency of the CY-WI that was originally established under pure waterlogging conditions.

In addition, another notable deficiency in current CY-WI research is disregarding the interactions among waterlogging events. Since waterlogging events usually occur many times across the whole cotton growing seasons, and cotton plants can adapt to waterlogging to a certain extent [24,61], this interaction effect deserves more considerations in future CY-WI research.

### 8.2. Regional-Scale CY-WI Relations

Nowadays, there have been many investigations that regionally related crop yields to waterlogging intensity, but relevant regional-scale analysis on cotton is still insufficient. Moreover, current regional CY-WI studies are mainly concentrated in the Middle-and-Lower Yangtze River region in China. However, for many other regions, regional-scale CY-WI relations can be very useful in ways differing from the field-scale CY-WI relations. Thus, regional CY-WI relations are expected to be established in more regions.

Previous attempts on regional CY-WI relations rely on meteorological indicators, severely disregarding the features of cotton plants. Therefore, it is necessary to propose novel regional indicators that can capture response features of cotton growth at different stages with precise time and spatial resolution.

### 8.3. Cotton Cultivars

To reduce the consequence of cotton waterlogging, especially in cotton-waterlogging-prone regions, it is necessary to use a waterlogging-tolerant variety. Cotton cultivars can greatly affect the CY-WI relations. Therefore, for important cotton areas, specific CY-WI should be determined by local data. In addition, with decades passed over, the dominating cotton variety in different cotton-producing regions has altered; thus, it is of practical importance to determine the CY-WI relations of new cultivars, thus assisting developing updated drainage schedules.

### 8.4. Initial and Late Cotton Growth Stages

As clearly shown in Figure 1c, the budding stage and the flowering and boll-forming stage contributed the majority of previous CY-WI experiments. This is understandable because these periods are crucial reproductive stages for cotton, during which cotton is sensitive to waterlogging conditions, as demonstrated by the results in Figure 4. Nevertheless, it should be noted that in many regions, cotton plants are objectively subjected to waterlogging stress from the seedling stage to the mature stage; thus, previous CY-WI relations are severely insufficient for these stages. As CY-WI relations are basis for drainage schedules, they are expected to be established for each stage. In future works, more CY-WI relations should be established for cotton seedling stage and boll opening stage.

### 8.5. Water Depths Under Waterlogging

The majority of previous CY-WI relation works employed fixed water depths and various waterlogging durations to establish different waterlogging treatments. In a few experiments, groundwater depths were regulated to achieve different levels of sub-surface waterlogging, but there is a rare study employing various surface water depths to obtain different levels of surface waterlogging. A primary reason for this phenomenon is the poor tolerance of cotton to waterlogging. The maximum surface water depth is about 20 cm in our literature collections, and according to our multiple experiences regarding cotton waterlogging experiments, deeper submergence depths may easily induce cotton crop failure through crop stem lodging. However, it should be cautious that due to the increase in extreme floods, waterlogging events with deep depths will be more common. Thus, various surface water depths, especially deep depths, are suggested to be considered in future CY-WI research.

### 8.6. Modern Tools Application

With the rapid development of science and technology, there are increasing modern tools that can be employed to improve CY-WI relation research. For instance, the AI technologies, especially machine-learning methods, have proven to be quite powerful in detecting non-linear relations between crops and environmental factors [81,82]. Considering that the existing CY-WI relations are usually simplified as linear forms (see previous Section 4), the AI methods can play key roles in establishing non-linear CY-WI relations. Second, remote sensing and satellite-derived data have been extensively used in monitoring crop growth status and soil water conditions. These data usually have higher time resolutions than the common climatic indicators, such as the SPEI and PA, which have been used in establishing regional CY-WI relations (see Figure 3) [83]. In addition, as indicated by a recent work [84], high-spatial-resolution satellite data can effectively map crop growth status under different water conditions; thus, these data can be used to dynamically map crop dry matter yields, which is the basis for developing dynamic CY-WI relations (see Equations (5) and (6)). Finally, it is also expected that the advanced sensors in fields can monitor real-time growth status of the stressed crops, thus determining dynamic CY-WI relations more accurately.

## 9. Conclusions

This review systematically examines previous research on CY-WI relations, and the most important findings are as follows:

(1) The reproductive stages of cotton, i.e., the flowering and boll-forming stage and the budding stage, receive the most attention; consequently, their CY-WI relations have been well established.
(2) Cotton waterlogging-relevant compound disasters/stresses usually affect CY-WI relations, but their influences differ greatly. Some can enhance the negative CY-WI relations, e.g., accompanying high temperatures, low temperatures, shade conditions, and soil salinity stress, while some others exhibit weakening effects, including prior-/post-drought or waterlogging.
(3) Recently, regional CY-WI relations are established by using cotton climatic yield and meteorological indicators. Moreover, they also verify the influence of compound disasters.

Actionable recommendations for future CI-WI research are as follows:

(1) More cotton waterlogging-relevant coupling stress, such as drought-waterlogging alternation and waterlogging-heat compound events, should be performed, accordingly adopting CY-WI relations to climate change.
(2) By using more high-quality regional data (e.g., climate data, crop yield and phases data, and remote sensing data), regional CY-WI relations should consider critical cotton growth features, such as cotton water requirements and waterlogging sensitivity at different stages.
(3) Future CY-WI relations are expected to establish for different waterlogging tolerance of cotton cultivars. In addition, for the key cotton-producing regions, specific CY-WI relations using representative cotton cultivars should be developed.
(4) More CY-WI relations for the initial and late phases of cotton should be well established, rather than only for reproductive stages.
(5) In future experiments, establishing CY-WI relations should account for gradient levels of high-depth submergence, rather than ideally assuming that only shallow surface water occurs.

## Figures and Tables

**Table 1:** Summary of key experimental works on CY-WI relations (including various levels of waterlogging intensities).

Authors	Study Region	Stress Establishment	Waterlogging Indicators	Yield Impact
Bange et al. (2004) [11]	Australia	Normal irrigations refer to 8 h irrigation for five times. Waterlogging treatments refer to extra 16-h irrigation at the third, fourth, and fifth irrigations in Experiment 1, refer to extra 44 h irrigation at first irrigation and extra 64 h irrigation at other four irrigations in Experiment 2, and refer to extra 64 h irrigation at all the five irrigations in Experiment 3.	Waterlogging duration (hours)	Experiment 1 exhibited no significant yield losses. Yield reduction rate was 37.6% (low ridge) in Experiment 2 and was 38.1% in Experiment 3.
Beegum et al. [15]	USA	At about 15 days of sowing, imposing waterlogging for 2, 4, 6, 8, 10, 12, and 14 days, respectively.	Waterlogging duration (days)	Two-day waterlogging has no significant impact. From 4-day waterlogging to 14-day waterlogging, yield reduction rate gradually increased from ~48% to ~80%.
Hodgson (1982) [34]	Australia	At each of the three irrigation timings (i.e., 20 December, 23 January, and 20 February), watering for 4, 8, 12, and 16 h, respectively.	Waterlogging duration (hours)	Cotton yields show significant negative relations to waterlogging hours. The determined CY-WI relation is: Cotton yield = 201.2 − 1.401 × waterlogging hours.
Kuai et al. (2015) [9]	China	On 66 days after transplanting seedlings, maintaining 1–2 cm of surface water for 3, 6, 9, or 12 days, respectively.	Waterlogging duration (days)	Three-, 6-, 9- and 12-day waterlogging events resulted in 16.0%, 24.1%, 39.5%, and 50.2% yield reduction, respectively.
Qian et al. (2021) [4]	China	In Experiment 1, at four cotton growth stages (seeding, budding, flowering, and boll opening), 3~6 days of surface waterlogging (0.1 m ponded water) and ~3–9 days of sub-surface waterlogging (perched water tables) were established by using an orthogonal experimental design. In Experiments 2 and 3, at three cotton growth stages (budding, flowering, and boll opening), ~1–5 days of surface waterlogging and ~3–8 days of sub-surface waterlogging were established.	Sum of excessive water tables above 30 cm groundwater depth (i.e., SFEW_30_)	In Experiment 1, the determined CY-WI relation is: Relative yield = 100 − 0.06 × Seeding SFEW_30_ − 0.11 × Budding SFEW_30_ − 0.10×Flowering SFEW_30_ − 0.07 × Boll-opening SFEW_30_ In Experiments 2 and 3, the determined CY-WI relations are: Relative yield = 100 − 0.06 × Budding SFEW_30_ − 0.09 × Flowering SFEW_30_ − 0.02 × Boll-opening SFEW_30_
Wang et al. (2017) [23]	China	At four growth stages (seeding, budding, flowering, and boll opening), five levels of waterlogging durations (2, 4, 6, 8, and 10 days) were established by keeping 5 cm surface water.	Waterlogging duration (days)	The highest yield reduction rates for the seeding, budding, flowering, and boll opening stages were 38.8%, 27.9%, 18.3%, and 7.6%, respectively.
Zhang et al. (2016) [22]	China	At three cotton growth stages (i.e., early squaring, peak flowering, and peak boll-setting stages), 10-day, 15-day, and 20-day waterlogging events (with 20 cm surface water) were established.	Waterlogging duration (days)	Yield reduction rates under 10-day, 15-day, and 20-day waterlogging events are: 53%, 59%, and 63%, respectively, at squaring; 27%, 37%, and 55%, respectively, at flowering; 13%, 15%, and 24%, respectively, at boll-setting.

**Table 2:** The influence of cotton variations on CY-WI relations.

Cotton Variations	Literature Sources	Effectiveness Description
Sicala V-2i and Nucotn 37.	Bange et al. (2004) [11]	In terms of waterlogging-induced yield losses, there were no significant differences in the two cultivars; thus, their results were averaged for analysis.
Thirteen upland cotton cultivars, i.e., Georgia King, McNair 1032, PD93057, LA 887, Codetec 401, DP 16, DP 90, Coker 315, CIM 443, Gohar 87, Sicot 71, Sicot 73, and Sicot 80, and one Pima cotton cultivar, i.e., Pima A-8.	Conaty et al. (2008) [10]	Under waterlogging stress, the minimum yields were observed in Pima A8 and Gohar 87, which were less than 700 kg ha^−1^. In comparison, the maximum yields were observed in Sicot 71 and Sicot 73, which were nearly 1600 kg ha^−1^.
Four tolerant cultivars, i.e., MNH-564, FH-114, MNH-786, and CIM-573, and 4 sensitive cultivars, i.e., N-KRISHMA, LRA-5166, CEDIX, and H-142	Hussain et al. (2018) [13]	The maximum yield was observed in a tolerant cultivar, i.e., MNH-786, which was 95.667 (g/plant). In comparison, the minimum yield was observed in a sensitive cultivar, i.e., CEDIX, which was 24.333 (g/plant).
CB-12, CB-13, Rupalli-1, and DM-3	Somaddar et al. (2023) [54]	Under severe waterlogging (9 days), the maximum yield reduction rate was observed in CB-13, which reached 62%. In comparison, the minimum yield reduction rate was observed in CB-12, which was as low as around 5%.
Six cotton cultivars from three cotton-producing regions. Two were from the Yangtze River valley, i.e., YZ1 and YZ2; two were from the Yellow River valley, i.e., YL1 and YL2; two were from the Northwest inland, i.e., NW1 and NW2.	Zhang et al. (2023) [55]	Under 7-day waterlogging stress, the maximum yield reduction rate was observed in NW 2, which was 22%. In comparison, the minimum yield reduction rate was observed in YZ 1, which was 6.9%.

**Table 4:** Comparison between the CY-WI relations at field scales and regional scales.

Study Scales	Waterlogging Indicators	Yield Indicators	Forms of CY-WI Relations	Waterlogging Growth Stages
Field scale	Waterlogging days and field water tables	Observed cotton yield data, or calculated yield losses.	Both linear and non-linear forms	Usually considering different growth stages (one or multiple stages).
Regional scale	Common meteorological indicators employed in drought and flooding research, such as the SPEI and PA.	Climate yield, which is also known as detrending yield. It is actually the part of cotton yield time series which is considered to be only affected by climatic factors.	Almost linear forms	In most cases, only considering the entire cotton growth period, regardless of growth stages.
